# Supplementary material for: Direct thermal charging cell for converting low-grade heat to electricity
Source: Nat Commun. 2019 Sep 12;10:4151. doi: 10.1038/s41467-019-12144-2 (PMC6742635; doi:10.1038/s41467-019-12144-2)
Supplement: Supplementary file 1 — Supplementary information [file 41467_2019_12144_MOESM1_ESM.pdf]

Supplementary Information

Direct thermal charging cell for converting low-grade heat to electricity

Wang et al.

Supplementary Movie

DTCC device demonstrations: first, commercial supercapacitor charged by a DTCC; second, smart window powered by six DTCCs; third, waste heat recovery from a running compressor to light up an OLED.

Supplementary Table 1 | Comparison of different TEC technologies for low grade heat to electricity conversion

Operating mode	***TEC system***	***Structure and Materials***	***α (mV/K)***	***η_E (η_E/η_{Carnot})***	***Advantages & Limitations***	***Ref***
Temperature gradient (continuous operation based on the temperature-dependent redox potentials at the hot and cold sides)	TGC	Electrode: Multi-walled carbon nanotubes(MWCNT) based electrode<br>Electrolyte: $K_3Fe(CN)_6/K_4Fe(CN)_6$	1.4	0.24% (1.4%)	Advantage: efficiency can be improved by using MWCNT instead of Pt electrodes.<br>Limitation: low efficiency caused by low electrolyte conductance	1
		Electrode: Carbon-based material<br>Electrolyte: $K_3[Fe(CN)_6]/(NH_4)_4[Fe(CN)_6]$ or $Fe_2(SO_4)_3/FeSO_4$	1.85	0.11% (0.4%)	Advantage: high power density by using improved electrolyte, electrolyte filled thermal separator and optimized carbon electrode materials.<br>Limitation: low efficiency	2
		Electrode: CNT aerogel sheets<br>Electrolyte: $K_3Fe(CN)_6/K_4Fe(CN)_6$	1.43	0.55% (3.95%)	Advantage: relative high efficiency and power density in TGCs<br>Limitation: low efficiency	3
Temperature gradient (flowing electrolytes in symmetric redox reactions at different temperatures)	RFB	Electrode: carbon cloth<br>Flow electrolyte: $[Fe(CN)_6]^{3-}/[Fe(CN)_6]^{4-}$ and V^{3+}/V^{2+}	3.0	1.8% (15%)	Advantage: relative high efficiency, high α, continuous operation across a broad range of temperatures<br>Limitation: relative high cost in electrolyte and operation	4
Temperature cycle (operating between hot and cold reservoirs alternating in a thermal cycle; charging and discharging at different temperatures)	TREC	Electrode: CuHCF and Cu<br>Electrolyte: $NaNO_3$ and $Cu(NO_3)_2$	1.2	3.7% (25%)	Advantages: high efficiency<br>Limitation: the need of external electricity for charging process and the use of ionic selective membrane	5
		Electrode: NiHCF and Ag/AgCl<br>Electrolyte: KCl	0.74	1.6% (13%)	Advantages: simple pouch cell structure without using ionic selective membrane<br>Limitation: the need of external electricity for charging process, relative low efficiency	6
		Electrode: $KFe^{II}Fe^{III}(CN)_6$ and $K_3Fe(CN)_6/K_4Fe(CN)_6$ with carbon cloth<br>Electrolyte: KNO_3	1.45	0.72% (6.0%)	Advantages: charging-free system<br>Limitation: low efficiency, the use of ionic selective membrane	7
	TRAB	Electrode: Cu<br>Electrolyte: $Cu(NO_3)_2/NH_4NO_3$	–	0.86% (6.1%)	Advantages: high power density, low cost<br>Limitation: ammonia stream is a serious concern regarding the leakage, stability, and safety	8
		Electrode: Cu<br>Flow electrolyte: $Cu(NO_3)_2/NH_4NO_3$	–	0.70% (5.0%)	Advantages: high power density, continuous operation<br>Limitation: ammonia stream is a serious concern regarding the leakage, stability, and safety	9
Temperature cycle (thermal-charging and discharging under high temperature and self-regenerated at low temperature)	DTCC	Electrode: GO/PtNPs and PANI<br>Electrolyte: $FeCl_2/FeCl_3$	5.0	3.52% (20%)	Advantages: both high efficiency and power density, high α, low cost and easy operation mode, simple pouch cell structure without using ionic selective membrane<br>Limitation: the degradation of long-term cycling	This work

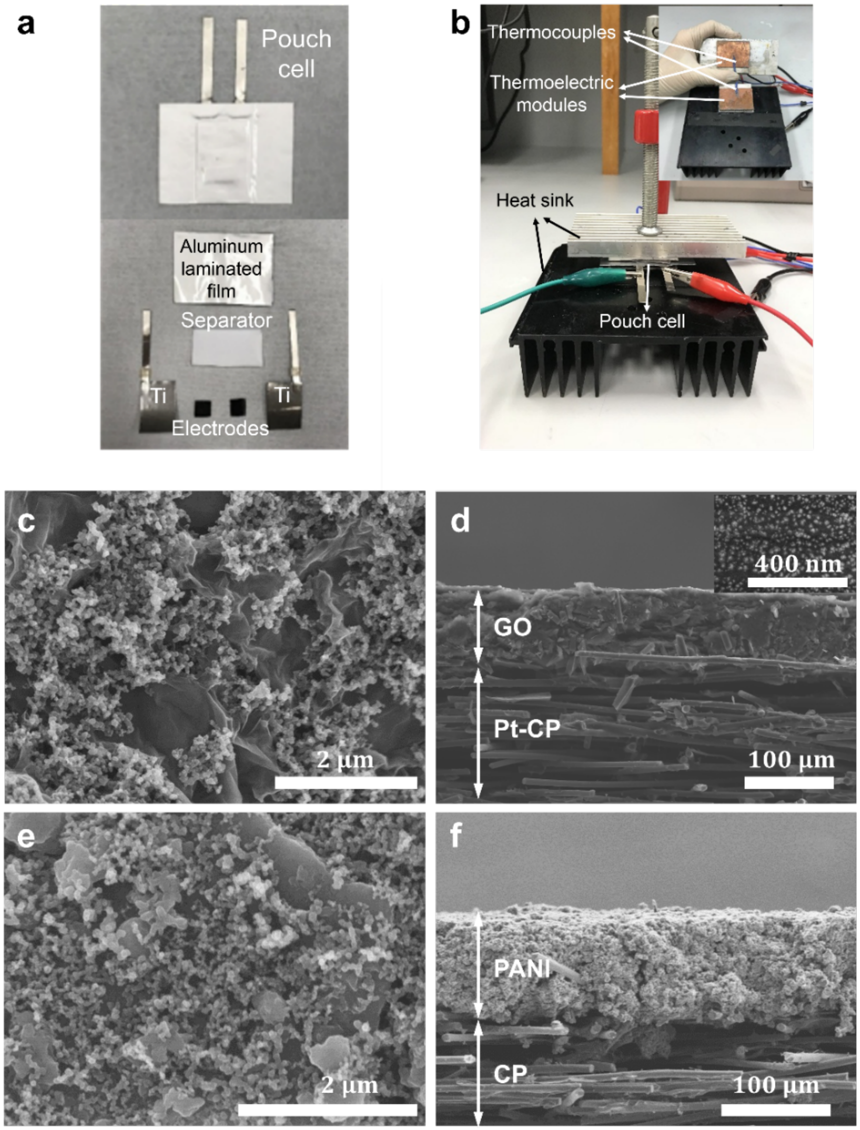

Supplementary Figure 1 | (**a**) Pouch cell configuration: GO/PtNPs cathode and PANI anode are assembled with titanium (Ti) foil current collector (connected to nickel tabs for sealing) and separated by the hydrophilic polypropylene separator. (**b**) Home-made temperature controller: pouch cell was located between two thermoelectric modules with heat sinks at each side, and thermocouples were placed between the pouch cell and TE modules. SEM images of (**c**) surface and (**d**) cross section (insert: Pt-coated carbon paper(Pt-CP)) of GO/PtNPs cathode. SEM images of (**e**) surface and (**f**) cross section of PANI anode. GO and PANI were both coated on the CP.

Supplementary Note 1: Thermodynamic principle of DTCC

Figure 1g illustrates the close-loop temperature-entropy diagram (T-S) of DTCC in one working cycle including thermal charging, electrical discharging and self-regeneration. During thermal charging, DTCC is heated up, inducing the fast faradic pseudocapacitive reactions at the GO-aqueous interface, where the protons attach to the GO to form a $GO\text{-}H^+$ layer. As mentioned in the main text, the isobaric heating and the chemisorption of protons on GO incorporating with the simultaneous reduction of Fe^{3+} to Fe^{2+} in GO/PtNPs cathode lead to an increase of entropy ($\Delta S_{1\rightarrow 2}$), generating a voltage. The adsorption of protons on the oxygen functional groups of GO would increase the number of molecules at the GO-aqueous interface, leading to a spatial entropy increase at the interfacial region. During electrical discharging, the cell V_{OC} can drive the oxidation of PANI to produce electrons through the external circuit and the electrons are carried by the reduction of Fe^{3+} at GO/PtNPs cathode concurrently. As this process is operated at a constant temperature, the overall entropy change $\Delta S_{2\rightarrow 3}$ is determined by the chemical reactions of electrode and electrolyte materials. In aqueous electrolyte, Fe^{3+} and Fe^{2+} can attract water molecules and gain the hydration shell as $[Fe(H_2O)_6]^{3+}$ and $[Fe(H_2O)_6]^{2+}$. The less charged Fe^{2+} core leads to a more disorder solvation shell, resulting in an increase of entropy during the reduction from Fe^{3+} to Fe^{2+} ($\Delta S_{Fe^{3+}\rightarrow Fe^{2+}} > 0$)[10]. Meanwhile, the oxidation of PANI conduces to an entropy increase due to the release of proton ($\Delta S_{PANI\rightarrow \text{Oxidized PANI}} > 0$) (Note: the reaction of PANI is illustrated in Supplementary Note 5). Therefore, $\Delta S_{2\rightarrow 3}$ increases as $\Delta S_{2\rightarrow 3} = \Delta S_{reaction} = \Delta S_{Fe^{3+}\rightarrow Fe^{2+}} + \Delta S_{PANI\rightarrow \text{Oxidized PANI}} > 0$. When cooled down to T_L, the $GO\text{-}H^+$ is turned to GO by the desorption of protons (refer to Supplementary Note 3 and Supplementary Figure 4), and the PANI and Fe^{3+} can be chemically regenerated (Supplementary Note 5). The isobaric cooling and negative reaction

entropy change (e.g., $\Delta S_{Fe^{2+} \rightarrow Fe^{3+}} < 0$, $\Delta S_{Oxidized\ PANI \rightarrow PANI} < 0$) result in a decrease of the overall entropy $\Delta S_{3 \rightarrow 1}$.

In a close-loop chemical cycle as shown in Fig. 1h, the net internal energy change is expected to be zero. And the total amount of work could be expressed as:

$$W = -\Delta G = \oint TdS \quad \text{(S1)}$$

Thus, the area of the cycle in T-S diagram represents the net output work of DTCC in a single run.

Supplementary Note 2: Open circuit voltage V_{OC} and temperature coefficient α

All cells were thermally charged under open circuit condition, and the V_{OC} can be expressed as[11],

$$V_{OC} = \Delta V_0 + \Delta V_{Anode} + \Delta V_{Cathode} \tag{S2}$$

where ΔV_0 is the difference between the electrochemical potentials of anode and cathode. ΔV_{Anode} and $\Delta V_{\text{Cathode}}$ are the change of anode and cathode potential, respectively, associating with temperature difference (ΔT) and α. The α can be defined as,

$$\alpha = \frac{\partial V}{\partial T} = \frac{V_H - V_L}{T_H - T_L} = \frac{\Delta S}{nF} \tag{S3}$$

where V_{H} and V_{L} is the voltage at T_H and T_L, respectively. ΔS and n are the entropy change and the number of electrons transferred in the reaction. In DTCC, the reactions in cathode including thermal-induced pseudocapacitance reaction of GO and thermogalvanic reaction of Fe^{2+}/Fe^{3+}, therefore the $\Delta V_{\text{Cathode}}$ is the sum of potential changes from the thermo-pseudocapacitive effect of GO and the thermogalvanic effect of Fe^{2+}/Fe^{3+}. The measured V_{OC} *versus* time of four cells, GO|KCl|Ti, GO|KCl|PANI, GO/PtNPs|KCl|PANI and GO/PtNPs|Fe^{2+}/Fe^{3+}|PANI, at different temperatures are shown in Supplementary Figure 2. The V_{OC} of GO|KCl|Ti cell is comparable to that of GO|KCl|PANI cell and GO/PtNPs|KCl|PANI, showing the similar α of 3.5 mV/K, 3.1 mV/K and 3.3 mV/K, respectively. With the aid of Fe^{2+}/Fe^{3+}, GO/PtNPs|Fe^{2+}/Fe^{3+}|PANI DTCC obtains the higher voltage compared to those of two cells at the same temperature and reaches a α of 5.0 mV/K, suggesting the occurrence of thermogalvanic effect between Fe^{2+}/Fe^{3+} and GO/PtNPs for further increased V_{OC}. The initial ΔV_0 of DTCC at room temperature is around 100 mV.

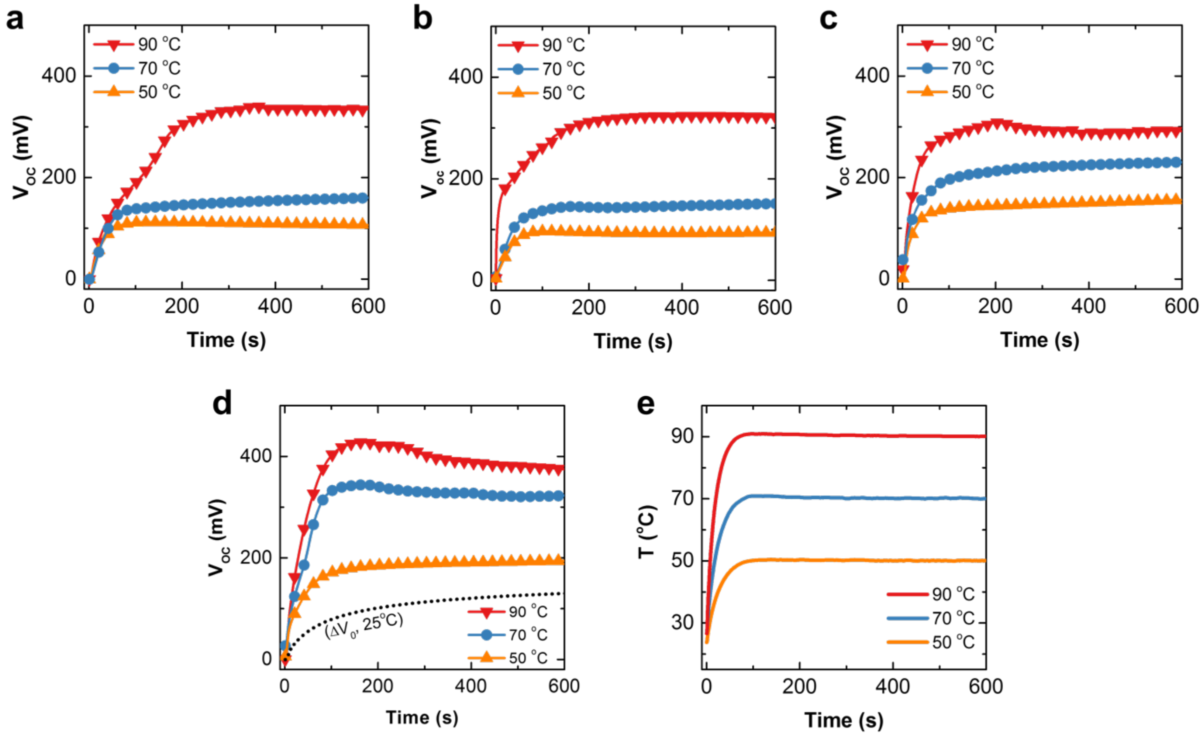

Supplementary Figure 2 | Open circuit voltages of (**a**) GO|KCl|Ti cell, (**b**) GO|KCl|PANI cell, (**c**) GO/PtNPs|KCl|PANI cell, and (**d**) GO/PtNPs| Fe^{2+}/Fe^{3+}|PANI DTCC measured at 50 ºC, 70 ºC and 90 ºC; the voltages reach stable values after 300s. Black dash line in **c** is the V_{OC} of DTCC at room temperature. (**e**) Measured temperature profiles of cells.

Supplementary Note 3: Calculation of electric double layer expansion at high temperature

We employ the Gouy-Chapman model to calculate the voltage contribution from the electric double layer (EDL) expansion at high temperature. To simply the calculation, we assume the electrode as a flat surface uniformly charged and ignore the complex surface geometry of the actual porous electrode. Poisson-Boltzmann equation could describe the system[12]:

$$\frac{d^2\phi}{dx^2} = -\frac{e}{\varepsilon\varepsilon_0}\sum_i n_i^0 z_i \exp\left(\frac{-z_i e\phi}{k_B T}\right) \tag{S4}$$

Where e is the elementary charge, ε is the dielectric constant, ε_0 is the vacuum permittivity, n_i^0 is the bulk concentration, z_i is the charge on ion i and k_B is the Boltzmann constant.

Derived from Gauss law, the surface charge density could be described as:

$$\sigma = \varepsilon\varepsilon_0\left(\frac{d\phi}{dx}\right)_{x=0} \tag{S5}$$

From equation S4 and S5, we obtain:

$$\sigma = (8k_B T\varepsilon\varepsilon_0 n^0)^{1/2}\sinh\left(\frac{ze\phi_0}{2k_B T}\right) \tag{S6}$$

During the expansion of EDL, the surface charge density of electrode in an open-circuit state is assumed remaining the same at different temperature, where we got:

$$\sigma_H = \sigma_L \tag{S7}$$

Applying equation S6 to S7, the voltage generated by EDL expansion could be obtained from:

$$(\phi_0)_H = \frac{2k_B T_H}{ze} arsinh\left[\left(\frac{\varepsilon_L T_L}{\varepsilon_H T_H}\right)^{\frac{1}{2}} \sinh\frac{ze(\phi_0)_L}{2k_B T_L}\right] \tag{S8}$$

Where $(\phi_0)_L$ equals to the built-in voltage ΔV_0 in our case and $\varepsilon(T) = 87.740 - 0.40008T + 9.398\times10^{-4}T^2 - 1.410\times10^{-6}T^3$ as in the reference[13].

Supplementary Note 4: Electrochemical impedance spectroscopy (EIS) for the study of temperature-induced pseudocapacitive effect at GO-aqueous interface

Electrochemical impedance spectroscopy (EIS) was conducted in the frequency range of 10^{-2}-10^{5} Hz to investigate the capacitive behavior of the GO-aqueous interface towards the temperature change, where a symmetric cell with two identical GO electrodes was used. Supplementary Figure 3a shows the Nyquist plot, where the inset presents that the charge transfer resistance (R_{CT}) corresponding to the first semi-circle (10^{5}-10^{2} Hz) decreases when temperature raises from 30 ^{o}C to 90 ^{o}C[14,15]. At the intermediate- to low-frequency range (10^{2}-10^{-1} Hz), the second incomplete semi-circle appears when the temperature is higher than 50 ^{o}C, evidencing the emergence of the faradaic surface reaction[15]. For the real part of the capacitance (C') (Supplementary Figure 3b), the C' increases with increasing temperature, representing an enhanced total capacitance at higher temperature. For the imaginary part of the capacitance (C") (Supplementary Figure 3c), the GO electrode becomes more frequency-dependent and C" increases sharply in the low-frequency range (10^{-1}-10^{-2} Hz) at higher temperatures, which indicates a resistive behavior different from the typical pure capacitive behavior[16]. The upward trend of C' and C" becomes more pronounced when increasing temperature, which can be attributed to an increased contribution from the faradic reactions. The findings described above lead us to believe that the temperature rise induces a fast and reversible faradaic process arising from the chemisorption of protons on the oxygen functional groups of the GO[17].

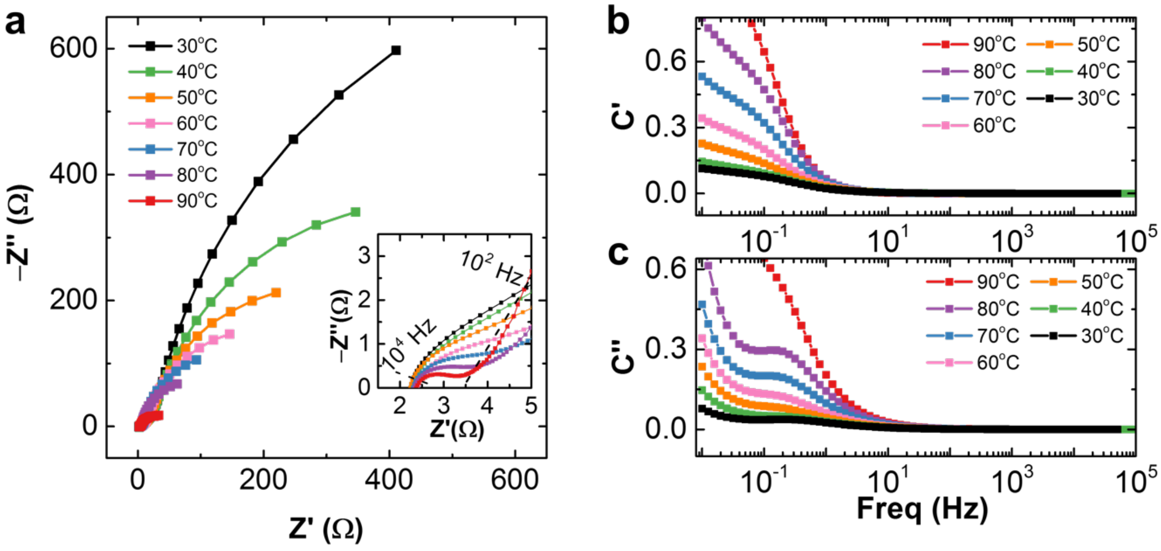

Supplementary Figure 3 | EIS of GO|KCl|GO symmetric cell at different temperatures. (**a**) Nyquist plot (insert: magnified figure in high-frequency region). Complex capacitance plots: (**b**) real capacitance and (**c**) imaginary capacitance versus frequency.

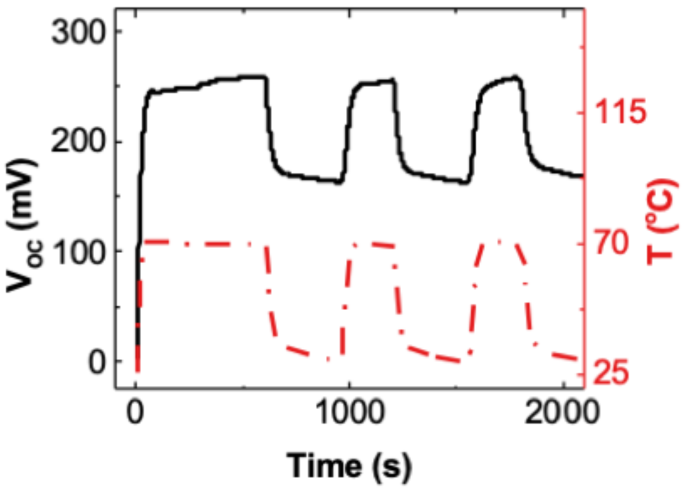

Supplementary Figure 4 | Voc and the temperature profile of GO|KCl|Ti cell when cycled between 25 °C and 70 °C.

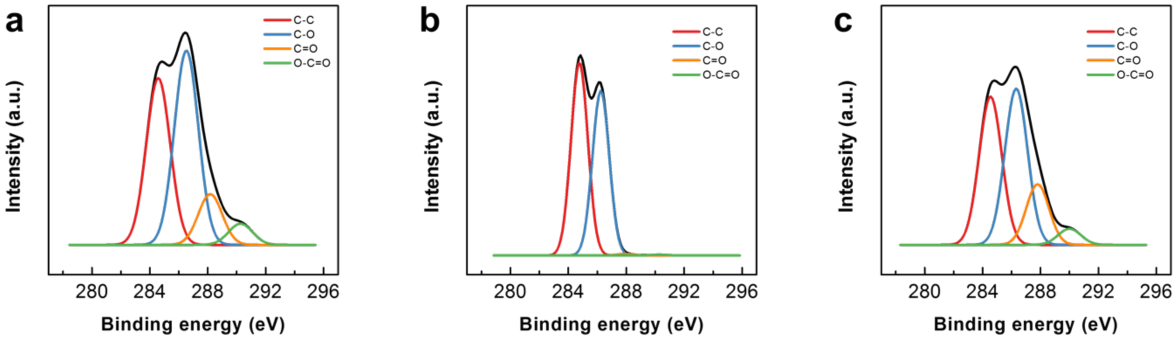

Supplementary Figure 5 | XPS of C1s at GO. (**a**) As-prepared GO sample with oxygen functional groups including C−C, C−O, C=O and O−C=O groups. (**b**) GO electrode after several thermal charging/electrical discharging cycles until no observation of thermal-induced voltage; the C=O and O−C=O could not be detected while the percentage of C−C is largely increased compared with **a**. (**c**) As-prepared GO after oxygen plasma treatment under 60W for 10s; the proportion of C=O is increased.

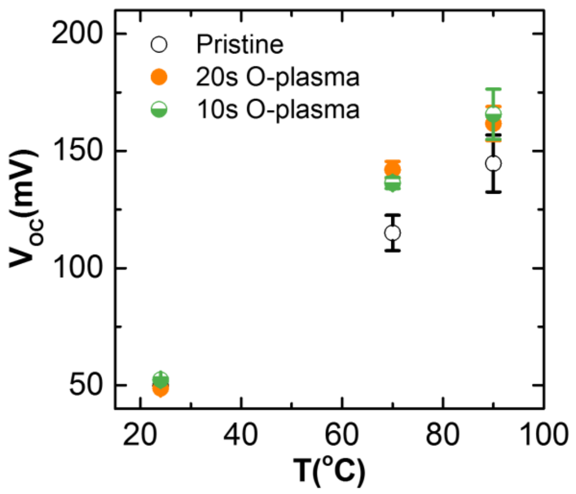

Supplementary Figure 6 | Open circuit voltage (Voc) of GO|KCl|Ti cells *versus* temperature, of which the GO cathodes were treated with/without oxygen plasmon. The GO|KCl|Ti cells using pretreated GO cathodes (oxygen plasma for 10s and 20s) show the increased Voc. Error bars were obtained from the standard errors of mean by independent samples.

Supplementary Note 5: Temperature-induced pseudocapacitive reactions at GO-aqueous interface

Here we chose GO as active electrode material because of its plenty of functionalities[18,19], of which oxygen functionalities (e.g., carbonyl C=O, carboxyl O−C=O) would induce a voltage due to their adsorption processes[20-22]. Supplementary Figure 7 shows TEM and Raman characterization of the as-prepared GO. GO shows high electron transparency in TEM and the ratio of D-band intensity to G-band intensity (I_D/I_G) of 0.908 indicates a defective crystal lattice.

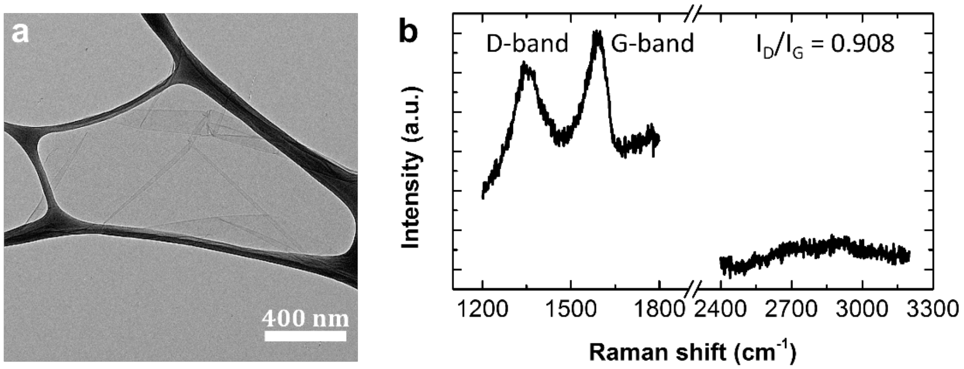

Supplementary Figure 7 | (**a**) TEM image and (**b**) Raman spectra of the as-prepared GO.

As mentioned in main text, we proposed that the thermal-induced voltage is attributed to the temperature-induced pseudocapacitive effect at the GO-aqueous interface. Previous research also supports our viewpoint that the increased temperature would enhanced the chemisorption of protons/ions on the modified carbon-based electrode, resulting in an increased capacitance[23,24]. The reactions of C=O and O−C=O functional groups in the GO dominates to the temperature-induced pseudocapacitance, which can be presented as[17,25],

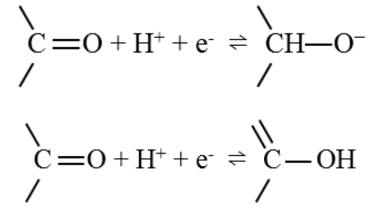

Supplementary Figure 8 shows the equivalent circuit of GO|KCl|PANI and GO/PtNPs|Fe^{2+}/Fe^{3+}|PANI DTCC at cathode-electrolyte interface. For GO|KCl|PANI cell, the pseudocapacitive behaviour of GO can be expressed by Randles circuit consisting of a parallel combination of pseudocapacitance (C_p) and Faradaic resistance (R_p) connected in series with a charge transfer resistance (R_{CT}) of KCl electrolyte. For GO/PtNPs|Fe^{2+}/Fe^{3+}|PANI DTCC cell, the Randles circuit of GO is connected to another Randles circuit inclusive of constant phase element (CPE) and R_{CT} of Fe^{2+}/Fe^{3+} redox electrolyte. The electrochemical redox reaction, such as $Fe^{2+/3+}$, rarely shows an ideal impedance response so that the CPE typically reflects a distribution of reactivity that is commonly represented in equivalent electrical circuits.

Open circuit thermal-charging process: When heating in open circuit condition, DTCC generates voltage via temperature-induced pseudocapacitive effect of GO and thermogalvanic effect of Fe^{2+}/Fe^{3+}, corresponding to C_p and CPE respectively. If we assume that the thermal-induced charge (Q_{charge}) is nearly fixed at the GO-aqueous interface at certain temperature, the capacitance would become less when C_p and CPE are connected in series, which causes the increase of voltage (Q_{charge}=CV).

Electrical discharging process: When external circuit is connected, the generated voltage can drive the oxidation of PANI so that the electrons are released from PANI anode to GO/PtNPs cathode. During discharging, the majority of electrons is provided by the oxidation of PANI, which can flow through cathode-electrolyte interface and are then carried by the reduction reaction of Fe^{3+} to Fe^{2+}. As compared with the large R_{CT} in GO|KCl|PANI cell, the redox-active electrolyte of Fe^{2+}/Fe^{3+} has a much lower R_{CT} to greatly facilitate the interfacial charge transfer and thus produces a much higher current. Therefore, the increase of total charge ($Q_{discharge} >> Q_{charge}$) significantly enhance the total discharge capacity during discharging process.

The thermal-induced voltage of DTCC is built by the thermo-pseudocapactive and the thermogalvanic effects connecting in series in open circuit condition, while the current of DTCC (driven by the voltage) are mainly sourced from the oxidation of PANI anode with the aid of the reduction reaction of Fe^{3+} to Fe^{2+} in electrolyte when the circuit is connected. Therefore, the total output current is not limited by the voltage source in cathode side because a large amount of electrons is provided by continuously oxidizing PANI anode after connecting the circuit.

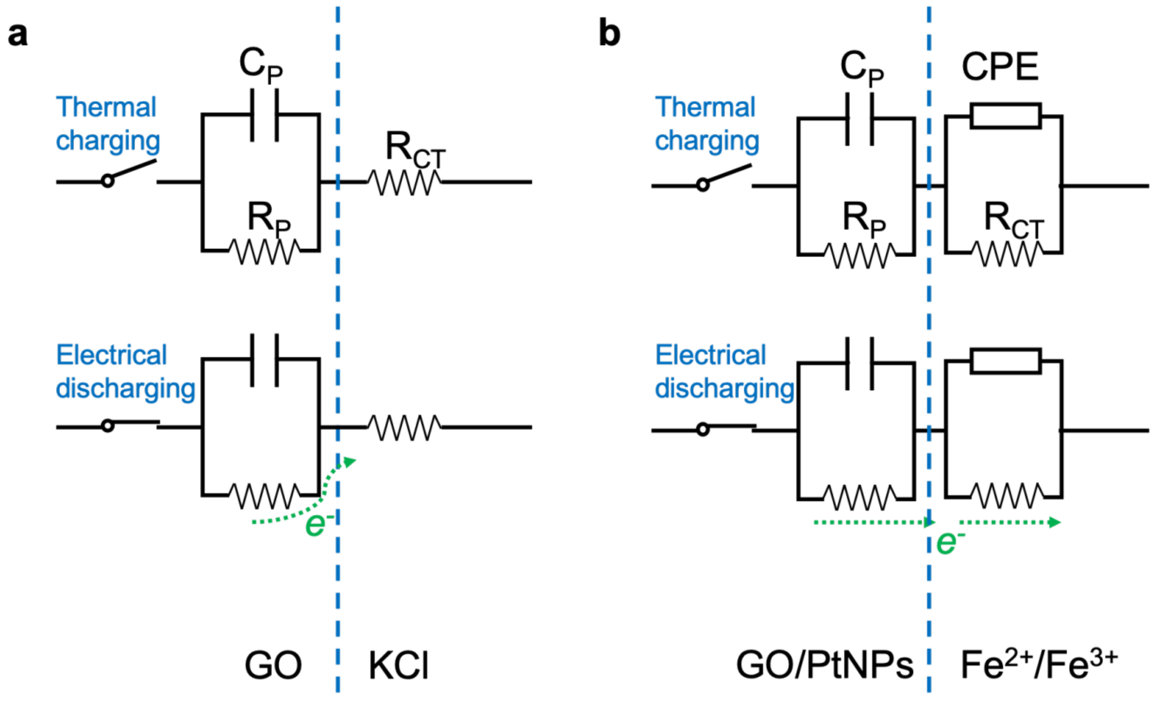

Supplementary Figure 8 | Equivalent circuit for (**a**) GO|KCl|PANI and (**b**) GO/PtNPs|Fe^{2+}/Fe^{3+}|PANI DTCC at cathode-electrolyte interface in thermal charging and electrical discharging process, where C_p is pseudocapacitance, R_p is a related faradic resistance of pseudocapacitive reaction, R_{CT} is charge transfer resistance between cathode and electrolyte and CPE stands for the constant phase element for Fe^{2+}/Fe^{3+} redox reaction.

Supplementary Note 6: Function of platinum nanoparticles (PtNPs)

PtNPs are vital in both thermal-charging and electrical discharging process. In thermal-charging process, the reduction reaction of Fe^{3+} happens under the catalysis of GO/PtNPs at T_H due to the positive α of Fe^{2+}/Fe^{3+}, which further enhances the V_{OC}. In the discharging process, with the aid of catalytic PtNPs, the Fe^{3+} effectively carries the electrons from GO/PtNPs cathode and is reduced to Fe^{2+}, which prevents the oxygen functional groups of GO from being reduced and consumed to retain the thermal-induced voltage and thus sustains the oxidation of PANI. Supplementary Figure 9 is the cyclic voltammetry (CV) for GO-coated carbon paper (GO-CP) and Pt-coated CP (Pt-CP) in the electrolyte of 0.5 M $FeCl_2/FeCl_3$. As compared with GO, PtNPs significantly enhance the catalytic ability for $Fe^{2+/3+}$ redox reactions.

Moreover, in another experiment, the GO/PtNPs electrode was immersed in 0.5 M $FeCl_3$ solution (Fe^{3+}-only electrolyte) and then heated to 50 ^{o}C for 2 min. Potassium ferricyanide solution ($K_3[Fe(CN)_6]$) can be used to detect Fe^{2+} in the solution. After adding few drops of 0.05 M $K_3[Fe(CN)_6]$ solution into the electrolyte with GO/PtNPs, the blue precipitate was formed and dispersed in the electrolyte so that the solution became a green color (Supplementary Figure 10), evidencing the existence of Fe^{2+} ions in the solution as Fe^{2+} ions would react with ferricyanide ions to form Turnbull's blue ($KFe[Fe(CN)_6]$) particles[26]. The control experiment (0.5 M $FeCl_3$ solution without GO/PtNPs was heated to 50 ^{o}C for 2 min) shows a brownish color due to the addition of $K_3[Fe(CN)_6]$ solution. The above experimental result provide evidence that GO/PtNPs would catalyze the reduction reaction of Fe^{3+} to Fe^{2+} when heating in open circuit condition. The possible chemical reaction is shown as below,

$$Fe^{3+} + {>}C{-}OH \xrightarrow{Pt} Fe^{2+} + {>}C{=}O + H^{+}$$

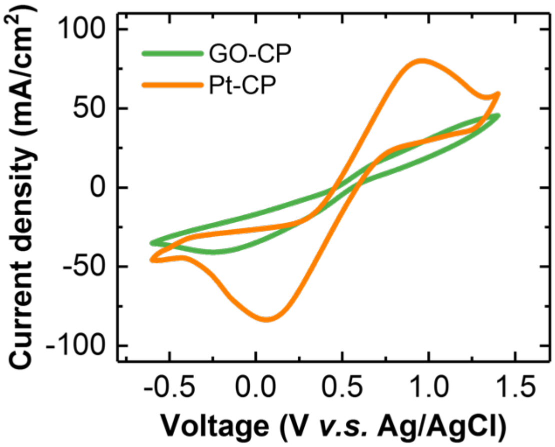

Supplementary Figure 9 | Cyclic voltammetry of Pt-CP and GO-CP in 0.5 M $FeCl_2/FeCl_3$ electrolyte. The scan rate is 50 mV s^{-1}.

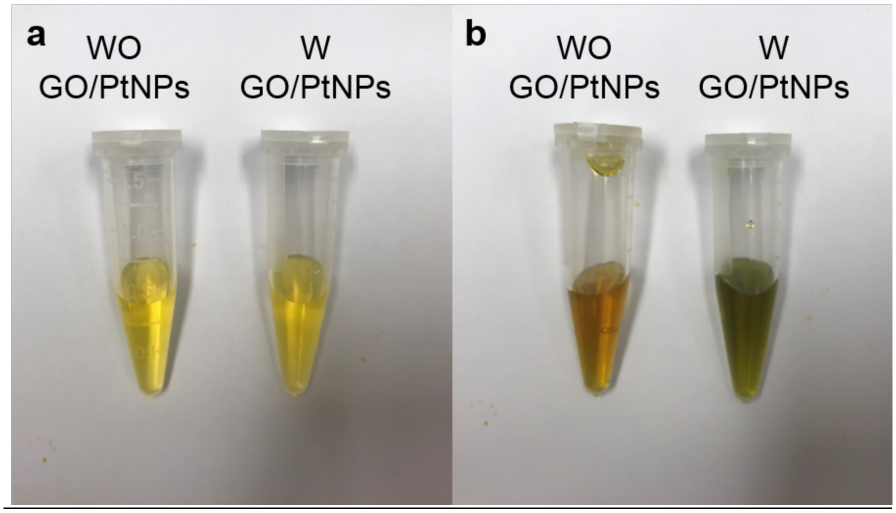

Supplementary Figure 10 | (**a**) 0.5 M FeCl3 solution with and without GO/PtNPs electrode after heating; (**b**) after adding 0.05 M $K_3[Fe(CN)_6]$ solution, the sample without GO/PtNPs shows a brownish color while that with GO/PtNPs turns to green color.

Supplementary Note 7: Chemical-regeneration of DTCC

In discharging process, the V_{OC} ranging from 0.3 to 0.4 V would drive the oxidation of PANI from protonated leucoemeraldine to emeraldine salt (ES) in the form of[27]:

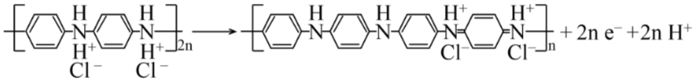

The oxidation of protonated leucoemeraldine PANI would release electrons and protons. Emeraldine PANI is in the transition redox states between leucoemeraldine PANI and pernigraniline PANI so that it is both cathodic and anodic electroactive[28]. It was reported that PANI-ES and Fe^{2+}/Fe^{3+} would easily react each other depend on pH value and temperature[29]. Particularly, PANI was reported to catalyze the oxidation of Fe^{2+} [30] and was transformed from oxidized state to reduced state in acidic environment[29,31].

After discharging of DTCC, there is a high concentration of Fe^{2+} in the acidic electrolyte. When cooled down to T_L, the Fe^{2+} is inclined to be oxidized to Fe^{3+} to reduce the entropy. Our system is therefore well-designed for the spontaneous chemical reaction of PANI-ES with a high concentration of Fe^{2+} and H^+ at T_L, which synergistically regenerates leucoemeraldine PANI and Fe^{3+} for the next cycle. The reaction can be seen as below:

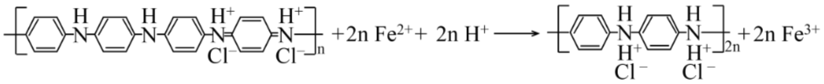

Supplementary Figure 11 shows the XPS characterization of the PANI samples for as-prepared, after-discharging and after-regeneration. XPS result of the as-prepared leucoemeraldine PANI shows the presence of nitrogen (N1s, ~400 eV), which consists of amine nitrogen (=N−, 398.8 eV), imine nitrogen (−NH−, 400 eV) and positively charged nitrogen ($-NH^+$, 402 eV). After discharging, the PANI was oxidized and thus −NH− and $-NH^+$ were transformed to =N−; the

percentage of −NH− decreased from 82% to 79% and the percentage of $-NH^+$ decreased from 9% to 8%, while the percentage of =N− increased from 9% to 13%. To regenerate the electrode, the used PANI (after discharging) was immersed in a 0.5 M $FeCl_2$/0.1 M HCl solution (similar to the condition of electrolyte after discharging) for 60 min. The content of −NH− increased to 83% and =N- decreased to 7%, evidencing the chemical reduction to recover the PANI. Meanwhile, the color of the solution was changed from green (Fe^{2+}) to yellow (Fe^{3+}) after the reaction.

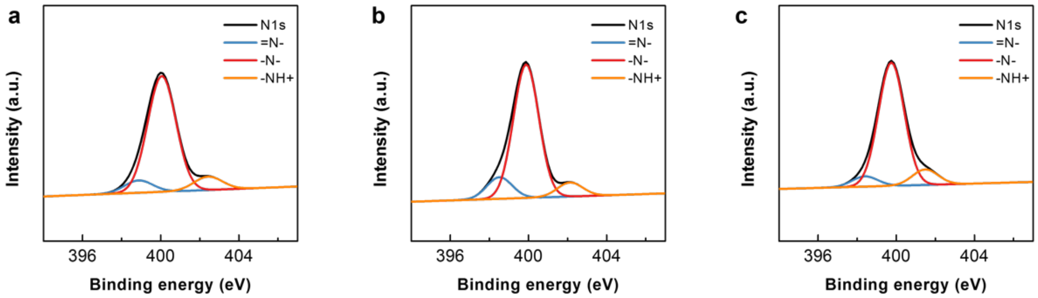

Supplementary Figure 11 | XPS of N1s on (**a**) as-prepared PANI, (**b**) PANI after one cycle of thermal charging and electrical discharging and **c**, Used PANI after immersing in 0.5 M $FeCl_2$/0.1 M HCl.

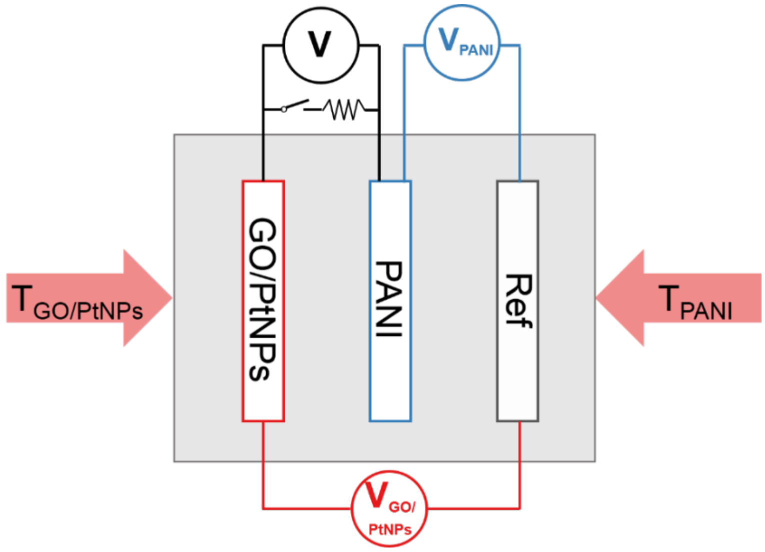

Supplementary Figure 12 | Schematic diagram for the measurement of DTCC. A three-electrode pouch cell with a Ti reference electrode is used to monitor the potential of each electrode independently.

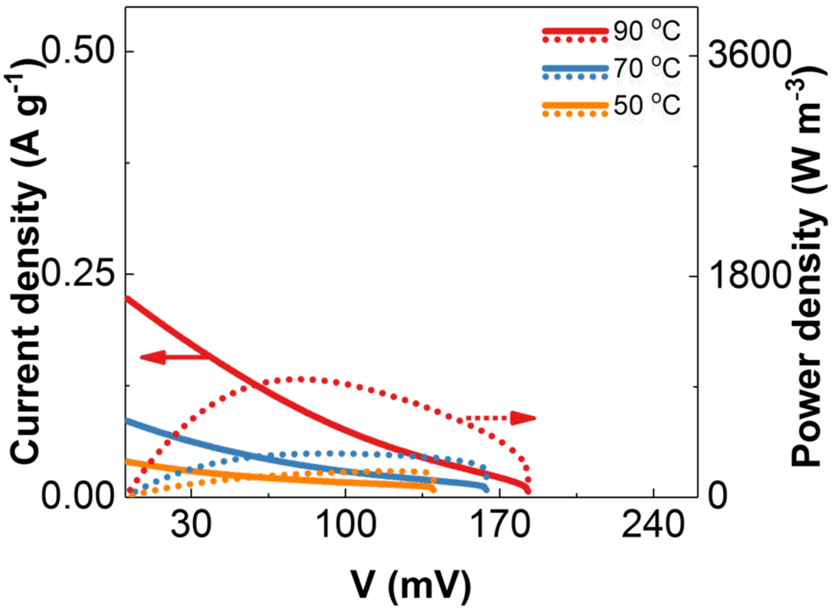

Supplementary Figure 13 | Current density-voltage (I-V) and volumetric power density-voltage (P-V) curves of GO|KCl|PANI cell, showing the maximal volumetric power densities of 213 W m^{-3}, 357 W m^{-3}, and 964 W m^{-3} at 50 °C, 70 °C, and 90 °C, respectively.

Supplementary Note 8: Fourier-transform infrared spectroscopy (FTIR) and observed problems on cyclability

The performance of DTCC was degraded by 20% after 20 cycles. Supplementary Figure 14 shows the Fourier-transform infrared spectroscopy (FTIR) of GO/PtNPs electrodes and PANI electrodes before cycling, after 10 cycles and after 20 cycles. GO/PtNPs electrodes and PANI electrodes remained unchanged in the first 10 cycles, but peaks were changed and shifted after 20 cycles. As described in XPS results of Supplementary Note 7, PANI is oxidized during electrical discharging and refreshed at room temperature via self-regeneration, which can be confirmed by the unchanged FTIR features after 10 cycle. The IR bands at 1568 cm^{-1} and 1494 cm^{-1} are assigned to C=C stretching vibrations of benzenoid and aromatic ring while that at 1634 cm^{-1} is attributed to C=N bond and C=C bond vibration[32]. After 20 cycles, the peak shift from 1568 cm^{-1} to 1634 cm^{-1} corresponds to a transformation from amine link to imine link of PANI, indicating the oxidation of PANI. Therefore, the PANI is still gradually oxidized and consumed after a long-term cycling. The peaks of GO become smaller due to the consumption of oxygen functional groups or the materials peeled off during cycles (refer to Supplementary Figure 15). One possible solution is to take the cell apart after tens of cycles and reassemble it after regenerating electrodes via chemical treatments[33,34].

Other than the problem of material degradation, in the current set-up, the cycling stability is also limited by the aqueous electrolyte, of which the large volume expansion and shrinkage during temperature cycles cause the active materials peeled off and the sealing problem (Supplementary Figure 15). The improvement of the cyclability of DTCCs can be expected with further optimization of electrode, electrolyte, and cell packaging.

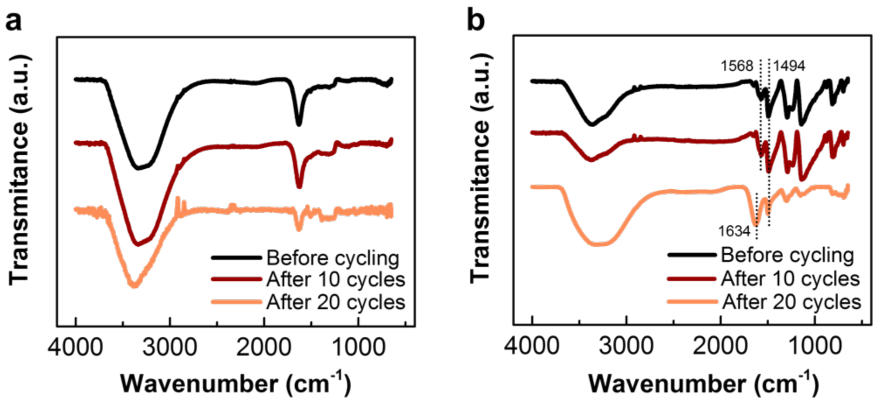

Supplementary Figure 14 | FTIR spectra of (**a**) GO/PtNPs electrodes and (**b**) PANI electrodes before cycling, after 10 cycles and after 20 cycles.

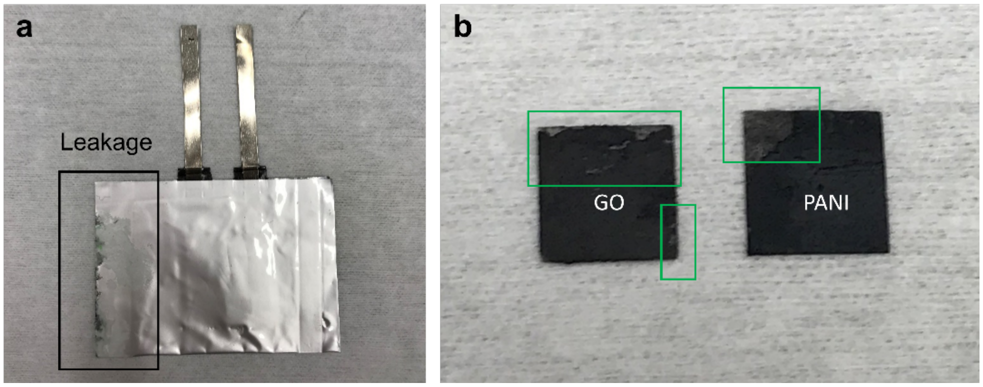

Supplementary Figure 15 | Observed problems in current set-up: (**a**) leakage of electrolyte and (**b**) formation of cracks on GO and PANI electrode.

Supplementary Note 9: Intermittent heat source

The DTCC is applicable to an intermittent heat source, where the V_{OC}, V_{Dis} and current density demonstrate good reproducibility towards on-off heating (Supplementary Figure 16a). The entire pouch cell is packaged using aluminum laminated film and the thickness is around 1-1.5 mm, which facilitates a rapid heating and cooling. The V_{OC} has a slight shift after the first cycle and keeps the same value in the following cycles (Supplementary Figure 16b). Basically, the reproducible thermal-induced voltage and current are obtained during intermittent heating/cooling cycles. The fast thermal response of the DTCC allows an immediate generation of voltage and electricity.

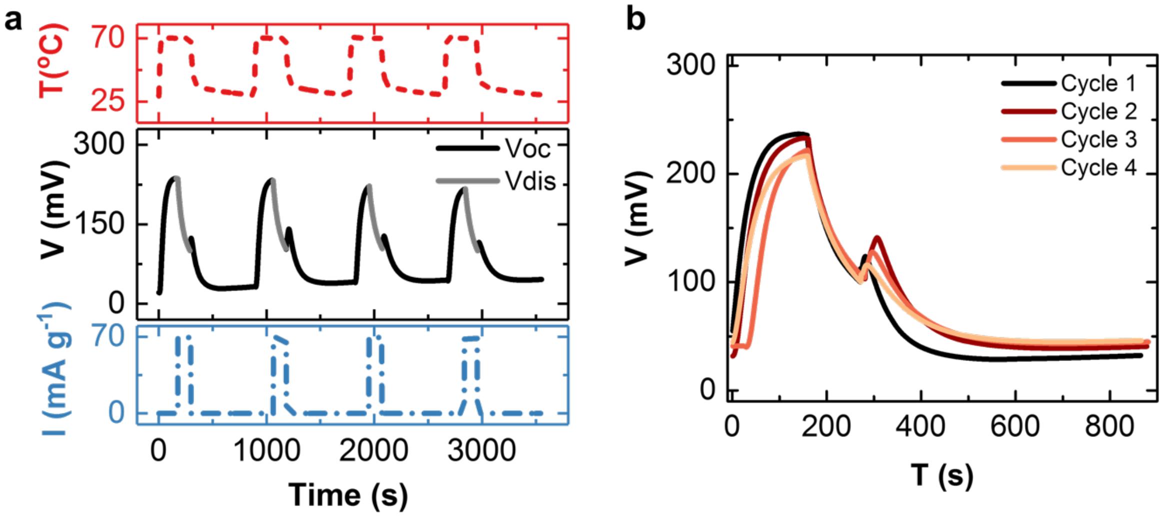

Supplementary Figure 16 | (**a**) Cell voltage and current *versus* time during intermittent heating/cooling cycles; (**b**) Overlapping of voltage-time curves for different cycles.

Supplementary Note 10: Efficiency calculation

The heat-to-electricity conversion efficiency (η_E) of DTCCs is estimated based on the ratio of output electrical work W to input thermal energy inclusive of the thermal energy Q_H absorbed for heating process and the continuous heat input Q_{iso} in discharging process at T_H, which can be expressed as,

$$\eta_E = \frac{W}{Q_H + Q_{iso}} \quad \text{(S9)}$$

DTCC has the same configuration as the normal electrochemical cell,

Negative electrode (−) | electrolyte | Positive electrode (+)

To heat up DTCC from T_L to T_H, the heat consumption is

$$Q_H = (1-\eta_{HX})\sum m_i C_{p,i} \Delta T \quad \text{(S10)}$$

where η_{HX} is the efficiency of heat recuperation, m_i and $C_{p,i}$ are the mass and specific heat capacity of the component i (active materals of GO/PtNPs, PANI and the electrolyte soaked in the sparator between electrodes are considered in our calculation), respectively. ΔT equals to $T_H - T_L$. During discharging, the energy consumption associated with the chemical reactions requires the continuous heat input to maintain the isothermal condition,

$$Q_{dis} = T_H \Delta S_H \quad \text{(S11)}$$

where T_H is the working temperature of DTCC, and the total entropy change ΔS_H equals to the summation of entropy changes at positive electrode ΔS_{H+} and negative electrode ΔS_{H-},

$$\Delta S_H = \int \Delta s_i \, dn_i \quad \text{(S12)}$$

where Δs_i and n_i are the partial molar entropy change and the amount of substance of the i th chemical, respectively. Deduced from equation S3, ΔS_H can be expressed as,

$$\Delta S_H = \int nF(\alpha_+ - \alpha_-)\, dn_i = \alpha \int nF\, dn_i = \alpha q_{dis} \tag{S13}$$

Applying equation S13 to S11,

$$Q_{dis} = \alpha q_{dis} T_H \tag{S14}$$

where q_{dis} is the amount of charges transferred during the discharging process. The discharging voltage V_{dis} and current I_{dis} (constant current) are recorded during the discharging time t. The capacitance (C) of the cell could be obtained from the linear part of discharging curve by

$$C = \frac{I_{dis} \times (t_2 - t_1)}{V_1 - V_2} \tag{S15}$$

The total net effective output work (W_{eff}) can be calculated from

$$W_{eff} = \int_{t(V_i)}^{t(\Delta V_0)} (V_{dis} - \Delta V_0) I_{dis} dt \approx \frac{1}{2} C (V_i - \Delta V_0)^2 \tag{S16}$$

Where V_i is the initial discharging voltage (after IR drop) and ΔV_0 is the built-in voltage of cell at the end of discharging. In our calculation, the W_{eff} excludes the work below the initial built-in voltage ΔV_0. The schematic diagram of discharging curve with related parameters is shown in Supplementary Figure 17.

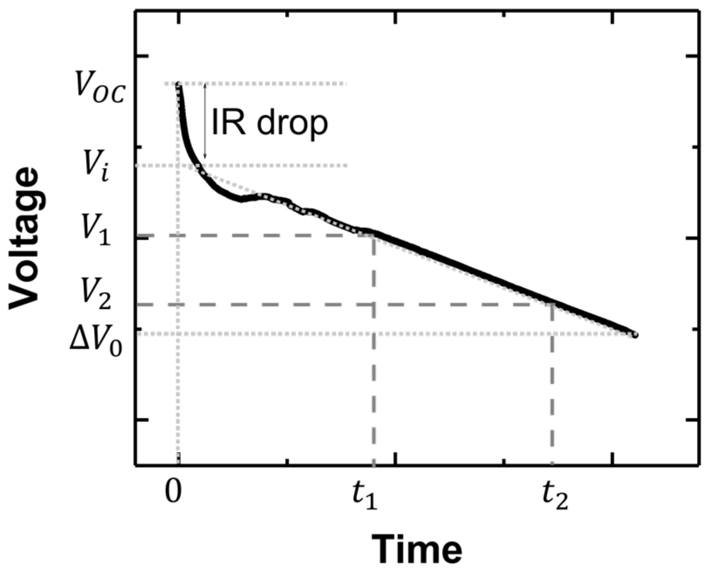

Supplementary Figure 17 | Schematic diagram of discharging curve.

Apply equation S10, S14 and S16 to S9, we obtain

$$\eta_E = \frac{W_{eff}}{(1 - \eta_{HX}) \sum m_i C_{p,i} \Delta T + \alpha q_{dis} T_H} \tag{S17}$$

All the parameters used in the calculation of η_E at 70 °C and 90 °C are shown in Supplementary Table 2 (without heat recuperation, $\eta_{HX} = 0$).

Supplementary Table 2 | Parameters for the efficiency calculation

**Parameters**	**Condition 1**	**Condition 2**	**Remarks**
T_L (°C)	25	25	
T_H (°C)	70	90	
η_{Carnot} (%)	13.12	17.91	
W (J)	0.089	0.21	Refer to Fig. 3b (70 °C) as example
Q_{dis} (C)	0.967	1.78	
α (mV K^{-1})	5.00	5.00	
m_{GO} (mg)	19.40	33.1	area: 1.5 cm x 1.0 cm
$C_{p(GO)}$ (J $g^{-1}K^{-1}$)	0.71	0.71	
m_{PANI} (mg)	6.53	5.15	area: 1.5 cmx 1.0 cm
$C_{p(PANI)}$ (J $g^{-1}K^{-1}$)	1.80	1.80	
$m_{Electrolyte}$ (mg)	2.25	2.68	Volume<br>(vol.):1.5cm×1.0cm(area)×25μm(thickness)<br>Porosity (p%): 50%<br>Density of electrolyte (d): 1.2 g mL^{-1}<br>$m_{Electrolyte}$ = vol. × p% × d
$C_{p(Electrolyte)}$ (J $g^{-1}K^{-1}$)	3.73	3.73	
Q_H (J)	1.53	2.68	
Q_{dis} (J)	1.66	3.23	
η_E (%)	2.80	3.52	
η_E /η_{Carnot} (%)	21.4	19.7	

Supplementary Note 11: η_E/η_{Carnot} versus gravimetric power density (W kg^{-1})

DTCCs demonstrate the high η_E/η_{Carnot} ratio *versus* gravimetric power density (W kg^{-1}) as compared with TREC (Supplementary Figure 18), which can reach 48 W kg^{-1} when operating at 90 ^{o}C. This gravimetric power density is comparable to the reported fuel cell systems[35].

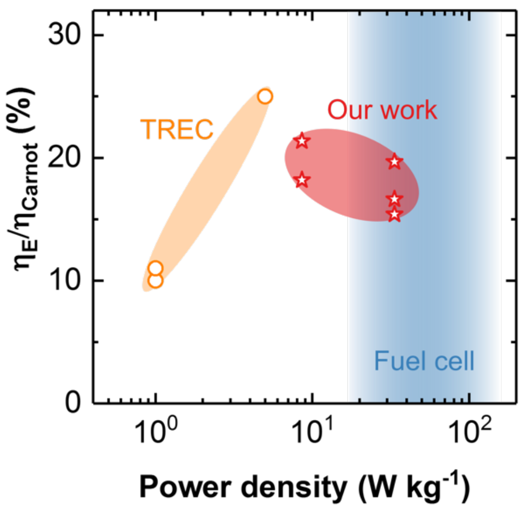

Supplementary Figure 18 | Ratio of η_E to η_{Carnot} *versus* gravimetric power density.

Supplementary Note 12: Commercial supercapacitor charged by DTCC

A commercial supercapacitor (Panasonic 4.7 F) can be charged to 0.15 V by using one DTCC at operating temperature of 67.4 ºC (Supplementary Figure 19). The DTCC was heated up from room temperature to 67.4 ºC at open circuit condition and the cell voltage reached 320 mV after 3min, and then it was connected to charge the supercapacitor. The easy combination of DTCC and energy storage devices makes the system design more convenient and flexible for various applications. The demonstration can be seen in Supplementary Movie 1.

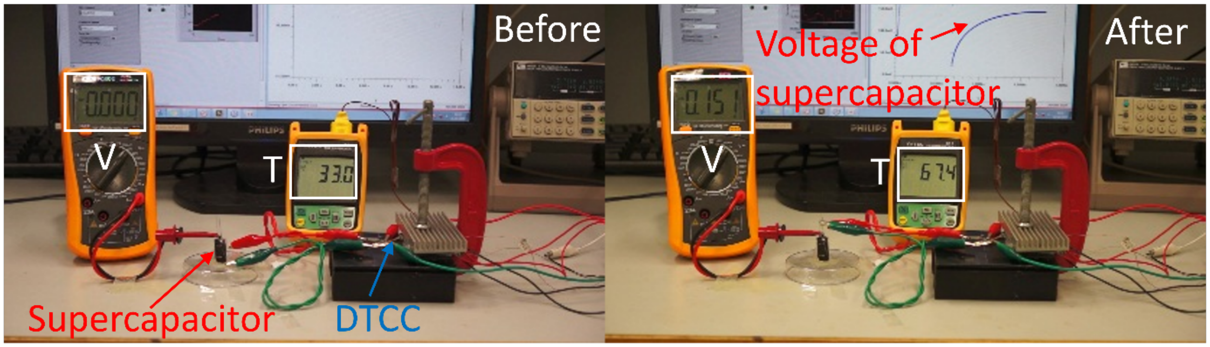

Supplementary Figure 19 | Demonstration of the commercial supercapacitor charged by DTCC.

Supplementary References

1. Hu, R. *et al.* Harvesting waste thermal energy using a carbon-nanotube-based thermo-electrochemical cell. *Nano Lett.* **10**, 838-846 (2010).
2. Zhang, L. *et al.* High Power Density Electrochemical Thermocells for Inexpensively Harvesting Low-Grade Thermal Energy. *Adv. Mater.* **29.12**, 1605652 (2017).
3. Im, H. *et al.* High-efficiency electrochemical thermal energy harvester using carbon nanotube aerogel sheet electrodes. *Nat. Commun.* **7**, 10600 (2016).
4. Poletayev, A. D., McKay, I. S., Chueh, W. C. & Majumdar, A. Continuous electrochemical heat engines. *Energy Environ. Sci.* **11**, 2964-2971 (2018).
5. Lee, S. W. *et al.* An electrochemical system for efficiently harvesting low-grade heat energy. . *Nat. Commun.* **5**, 3942 (2014).
6. Yang, Y. *et al.* Membrane-free battery for harvesting low-grade thermal energy. *Nano Lett.* **14**, 6578-6583 (2014).
7. Yang, Y. *et al.* Charging-free electrochemical system for harvesting low-grade thermal energy. *Proc. Natl. Acad. Sci.* **111**, 17011-17016 (2014).
8. Zhang, F., Liu, J., Yang, W. & Logan, B. E. A thermally regenerative ammonia-based battery for efficient harvesting of low-grade thermal energy as electrical power. *Energy Environ. Sci.* **8**, 343-349 (2015).
9. Zhu, X., Rahimi, M., Gorski, C. A. & Logan, B. A Thermally-Regenerative Ammonia-Based Flow Battery for Electrical Energy Recovery from Waste Heat. *ChemSusChem* **9**, 873-879 (2016).
10. Huang, B. *et al.* Non-covalent interactions in electrochemical reactions and implications in clean energy applications. *Phys. Chem. Chem. Phys.* **20.23**, 15680-15686 (2018).

11. Wang, J. *et al.* "Thermal Charging" Phenomenon in Electrical Double Layer Capacitors. *Nano Lett.* **15**, 5784-5790 (2015).

12. Bard, A. J., Faulkner, L. R., Leddy, J. & Zoski, C. G. *Electrochemical methods: fundamentals and applications*. Vol. 2 (wiley New York, 1980).

13. Malmberg, C. & Maryott, A. Dielectric Constant of Water from 0 ^{o}C to 100 ^{o}C. *Journal of research of the National Bureau of Standards*. **56.1**, 1 (1956).

14. Augustyn, V., Simon, P. & Dunn, B. Pseudocapacitive oxide materials for high-rate electrochemical energy storage. *Energy Environ. Sci.* **7**, 1597-1614 (2014).

15. Gaikar, P. *et al.* Pseudocapacitive performance of a solution-processed β-Co (OH) 2 electrode monitored through its surface morphology and area. *Dalton Trans.* **46**, 3393-3399 (2017).

16. Conway, B. E. *Electrochemical supercapacitors: scientific fundamentals and technological applications*. (Springer Science & Business Media, 2013).

17. Fang, Y. *et al.* Renewing Functionalized Graphene as Electrodes for High-Performance Supercapacitors. *Adv. Mater.* **24**, 6348-6355 (2012).

18. Zhao, J., Liu, L. & Li, F. *Graphene oxide: physics and applications*. (Springer, 2015).

19. Zhai, P. *et al.* Tuning Surface Wettability and Adhesivity of a Nitrogen-Doped Graphene Foam after Water Vapor Treatment for Efficient Oil Removal. *Adv. Mater. Interfaces* **2**, 1500243 (2015).

20. Fontecha-Cámara, M., López-Ramón, M., Alvarez-Merino, M. & Moreno-Castilla, C. About the endothermic nature of the adsorption of the herbicide diuron from aqueous solutions on activated carbon fiber. *Carbon* **11**, 2335-2338 (2006).

21. Lim, H., Lu, W., Chen, X. & Qiao, Y. Anion size effect on electrode potential in a nanoporous carbon. *Int. J. Electrochem. Sci* **7**, 2577-2583 (2012).

22. Lim, H., Zhao, C. & Qiao, Y. Performance of thermally-chargeable supercapacitors in different solvents. *Phys. Chem. Chem. Phys.* **16**, 12728-12730 (2014).

23. Zhao, F., Liang, Y., Cheng, H., Jiang, L. & Qu, L. Highly efficient moisture-enabled electricity generation from graphene oxide frameworks. *Energy Environ. Sci.* **9**, 912-916 (2016).

24. Masarapu, C., Zeng, H. F., Hung, K. H. & Wei, B. Effect of temperature on the capacitance of carbon nanotube supercapacitors. *ACS Nano* **3**, 2199-2206 (2009).

25. Chen, C.-M. *et al.* Hierarchically aminated graphene honeycombs for electrochemical capacitive energy storage. *J. Mater. Chem.* **22**, 14076-14084 (2012).

26. Izatt, R. M., Watt, G. D., Bartholomew, C. H. & Christensen, J. J. Calorimetric study of Prussian blue and Turnbull's blue formation. *Inorg. Chem.* **9**, 2019-2021 (1970).

27. Kaner, R. Polyaniline, a novel conducting polymer. *J. Chem. Soc. Faradat Trans.* **82**, 2385 (1986).

28. Noufi, R., Nozik, A. J., White, J. & Warren, L. F. Enhanced stability of photoelectrodes with electrogenerated polyaniline films. *J. Electrochem. Soc.* **129**, 2261-2265 (1982).

29. Nicolau, Y.-F. & Beadle, P. (Google Patents, 2001).

30. Kar, P. *Doping in conjugated polymers*. (John Wiley & Sons, 2013).

31. Ren, L. *et al.* High capacitive property for supercapacitor using Fe^{3+}/Fe^{2+} redox couple additive electrolyte. *Electrochim. Acta* **231**, 705-712 (2017).

32. Butoi, B., Groza, A., Dinca, P., Balan, A. & Barna, V. Morphological and structural analysis of polyaniline and poly (o-anisidine) layers generated in a DC glow discharge

plasma by using an oblique angle electrode deposition configuration. *Polymers* **9**, 732 (2017).

33. Moon, D. K., Ezuka, M., Maruyama, T., Osakada, K. & Yamamoto, T. Chemical reduction of the emeraldine base of polyaniline by reducing agents and its kinetic study. *Die Makromolekulare Chemie: Macromolecular Chemistry and Physics* **194**, 3149-3155 (1993).
34. Zhao, H. *et al.* Oxygen plasma-treated graphene oxide surface functionalization for sensitivity enhancement of thin-film piezoelectric acoustic gas sensors. *ACS Appl. Mater. Interfaces* **9**, 40774-40781 (2017).
35. Wachsman, E. D. & Lee, K. T. Lowering the temperature of solid oxide fuel cells. *Science* **334**, 935-939 (2011).
